# Haustorium Inducing Factors for Parasitic Orobanchaceae

**DOI:** 10.3389/fpls.2019.01056

**Published:** 2019-08-29

**Authors:** Vincent Goyet, Syogo Wada, Songkui Cui, Takanori Wakatake, Ken Shirasu, Gregory Montiel, Philippe Simier, Satoko Yoshida

**Affiliations:** ^1^Laboratoire de Biologie et Pathologie Végétales, Université de Nantes, Nantes, France; ^2^Division of Biological Science, Graduate School of Science and Technology, Nara Institute of Science and Technology, Ikoma, Nara, Japan; ^3^Institute for Research Initiatives, Division for Research Strategy, Nara Institute of Science and Technology, Ikoma, Nara, Japan; ^4^RIKEN Center for Sustainable Resource Science, Yokohama, Japan; ^5^Graduate School of Science, The University of Tokyo, Tokyo, Japan

**Keywords:** haustorium, haustorium-inducing factor, Orobanchaceae, parasitic plants, Striga, quinone, cytokinin, lignin

## Abstract

Parasitic plants in the Orobanchaceae family include devastating weed species, such as *Striga*, *Orobanche*, and *Phelipanche*, which infest important crops and cause economic losses of over a billion US dollars worldwide, yet the molecular and cellular processes responsible for such parasitic relationships remain largely unknown. Parasitic species of the Orobanchaceae family form specialized invasion organs called haustoria on their roots to enable the invasion of host root tissues. The process of forming haustoria can be divided into two steps, prehaustorium formation and haustorium maturation, the processes occurring before and after host attachment, respectively. Prehaustorium formation is provoked by host-derived signal molecules, collectively called haustorium-inducing factors (HIFs). Cell wall-related quinones and phenolics have been known for a long time to induce haustoria in many Orobanchaceae species. Although such phenolics are widely produced in plants, structural specificities exist among these molecules that modulate their competency to induce haustoria in different parasitic plant species. In addition, the plant hormone cytokinins, structurally distinct from phenolic compounds, also trigger prehaustorium formation in Orobanchaceae. Recent findings demonstrate their involvement as rhizopsheric HIFs for *Orobanche* and *Phelipanche* species and thus address new activities for cytokinins in haustorium formation in Orobanchaceae, as well as in rhizospheric signaling. This review highlights haustorium-inducing signals in the Orobanchaceae family in the context of their host origin, action mechanisms, and species specificity.

## Introduction

Limitations in soil fertility have influenced the diversification of nutrient acquisition strategies in plants (Zemunik et al., 2015). Parasitic plants develop haustoria (singular haustorium), specialized organs for nutrient acquisition from host plants (Yoshida et al., 2016). Although the term haustoria is commonly used in biotrophic plant-pathogenic fungi or oomycetes and both fungi and parasitic plant haustoria have functions for nutrient withdrawing, the morphology and organization of haustoria in these organisms are quite different. The fungal haustoria represent unicellular hyphae surrounded by host-derived extrahaustorial membrane, whereas haustoria of parasitic plants are multicellular organs that consist of different cell types and invade host tissues intercellularly (Yoshida et al., 2016). Among angiosperms, there are approximately 4,500 species of parasitic plants that have the ability to form haustoria and connect their vasculatures with those of their hosts to obtain water and nutrition. Parasitic plants have evolved independently at least 12 times and can be classified into two groups, root parasites and stem parasites, depending on which host tissue is parasitized (Westwood et al., 2010). The Orobanchaceae family contains the largest number of parasitic plants and consists of root parasites with various degrees of host dependency and photosynthetic activity, i.e., facultative parasites, obligate hemiparasites, and obligate holoparasites. Facultative parasites include species that have the ability for an autotrophic lifestyle and opportunistically parasitize host plants. Obligate parasites include species that cannot complete their life cycles without host plants; those who have photosynthetic activity are called hemiparasites, and those who have lost photosynthetic ability are called holoparasites (Westwood et al., 2010). Some obligate parasitic plants in the Orobanchaceae family, especially in the genera of *Striga*, *Orobanche*, and *Phelipanche*, parasitize crop species and cause devastating yield losses. *Striga* species infect maize, sorghum, and upland rice especially in sub-Saharan Africa with the economic damage caused by *Striga* estimated to be about 1 billion US dollars per year (Spallek et al., 2013). *Orobanche and Phelipanche* species infect economically important crops including pea, faba bean, sunflower, and oilseed rape (Fernández-Aparicio et al., 2016a). Germination of these obligate parasites depends on stimulating compounds including strigolactones and strigolactone-like compounds exuded by the roots of host plants (Yoneyama et al., 2010). Signaling components involved in strigolactone perception in Orobanchaceae are found to be associated with their germination (Lechat et al., 2015; Brun et al., 2018; Brun et al., 2019). In comparison, the molecular basis underlying haustorium formation remains mostly unknown. Nevertheless, recent studies have identified several haustorium-inducing chemicals for various Orobanchaceae members and have characterized their structural commonality and species specificity. This review will focus on the early developmental processes for haustorium formation in the Orobanchaceae family.

## Haustorium Developmental Processes

According to the parasite species, two kinds of haustoria can be observed in the Orobanchaceae family: one is a “terminal haustorium” that develops on the radicle tip, and the other is a “lateral haustorium” that develops as a lateral extension of primary and lateral roots of parasitic plants (Joel, 2013; Yoshida et al., 2016). Most facultative parasites in the Orobanchaceae form lateral haustoria, while terminal haustoria are characteristic of several obligate holo- and hemiparasite clades, such as *Orobanche*, *Phelipanche*, *Striga*, and *Alectra* (Joel, 2013). Despite the structural variation among haustoria, haustorial developmental processes seem largely conserved among species.

**Figure 1:**
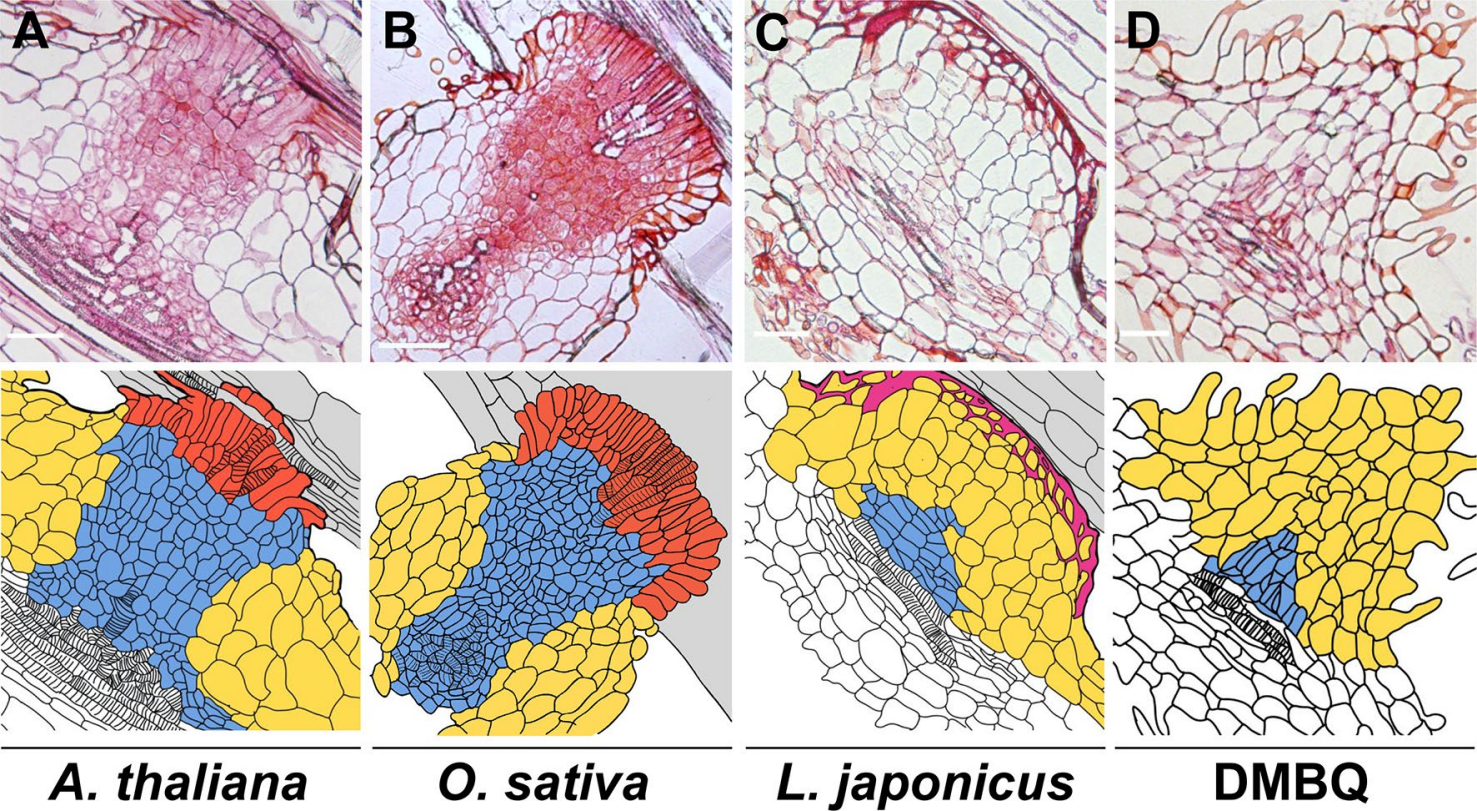
**Haustorial cell structures formed upon infection of various plants or induced by DMBQ.** The upper photos show the longitudinal sections of *P. japonicum* haustoria after infection of susceptible plants (*Arabidopsis thaliana* **(A)** and *Oryza sativa* **(B)**) and a resistant plant (*Lotus japonicus* **(C)**) or induction by DMBQ treatment **(D)**. The lower drawings show the cell outlines of the upper sections. The colors denote cell types. Red, blue, yellow, purple, and gray indicate intrusive cells, small (procambium-like) cells, large cells, lignin accumulation, and host root cells, respectively. Stripes mark the location of xylem cells. Bars = 100 µm.

Haustorium formation processes can be divided into two phases: prehaustorium formation induced by haustorium-inducing factors (HIFs) and haustorium maturation upon host infection. Except for some species that can form self-haustoria (Xiang et al., 2018), the initial step of haustorium formation is provoked by host-derived small compounds, collectively called HIFs. Several hours after recognition of HIFs, parasite roots or radicles start to show morphological changes including cell expansion, cell division, and differentiation of haustorial hairs in hemiparasites or papillae in holoparasitic genera *Orobanche* and *Phelipanche* spp., and semi-spherically shaped prehaustorial organs are formed within a few days (Riopel and Musselman, 1979; Baird and Riopel, 1985; Cui et al., 2016; Ishida et al., 2016; Goyet et al., 2017). The resultant organ is defined as “prehaustorium” or an “early haustorial structure” due to its premature architecture. Haustorium maturation occurs only after host attachment and is not solely promoted by HIFs, indicating the requirement of additional host signal(s) other than HIFs for structural maturation. Once the haustorium reaches host tissues, the epidermal cells of the parasite haustorium apex differentiate into intrusive cells, the specialized cells for host invasion with characteristic elongated shapes (**Figure 1**) (Stephens, 1912; Olivier et al., 1991). Intrusive cells grow inside host tissues toward host vasculatures. Upon reaching the host vasculature, some of the adjacent parasite cells differentiate into tracheary elements and form a xylem connection between the host and the parasite (Dorr, 1997), a structure called xylem bridge. Cell lineage analysis using the facultative parasite *Phtheirospermum japonicum* revealed dynamic cell fate transitions during haustorium formation (Wakatake et al., 2018). Intrusive cells originate from epidermal cells, and the central part of the haustorium has small cells with procambium-like cell identity as indicated by marker gene expression (Wakatake et al., 2018). Notably, development of such internal structures is only observed concomitant with host interaction; some incompatible hosts can interfere with the formation of intrusive cells and the subsequent xylem bridge (**Figure 1**) (Olivier et al., 1991; Yoshida and Shirasu, 2009), rendering these cells minimal prerequisites for establishing parasitism. Transcriptome analysis of *P. japonicum* revealed that *YUCCA3*, a gene encoding a key enzyme for auxin biosynthesis, is induced by HIF treatment in the epidermal cell layer of the haustorium-forming site (Ishida et al., 2016). Artificial induction of *YUCCA3* expression within epidermal cells of *P. japonicum* roots can induce haustorium-like semi-spherical structures with haustorial hair proliferation (Ishida et al., 2016), indicating the involvement of auxin biosynthesis in organogenesis of the prehaustorium.

## HIFs for Hemiparasites

**Figure 2:**
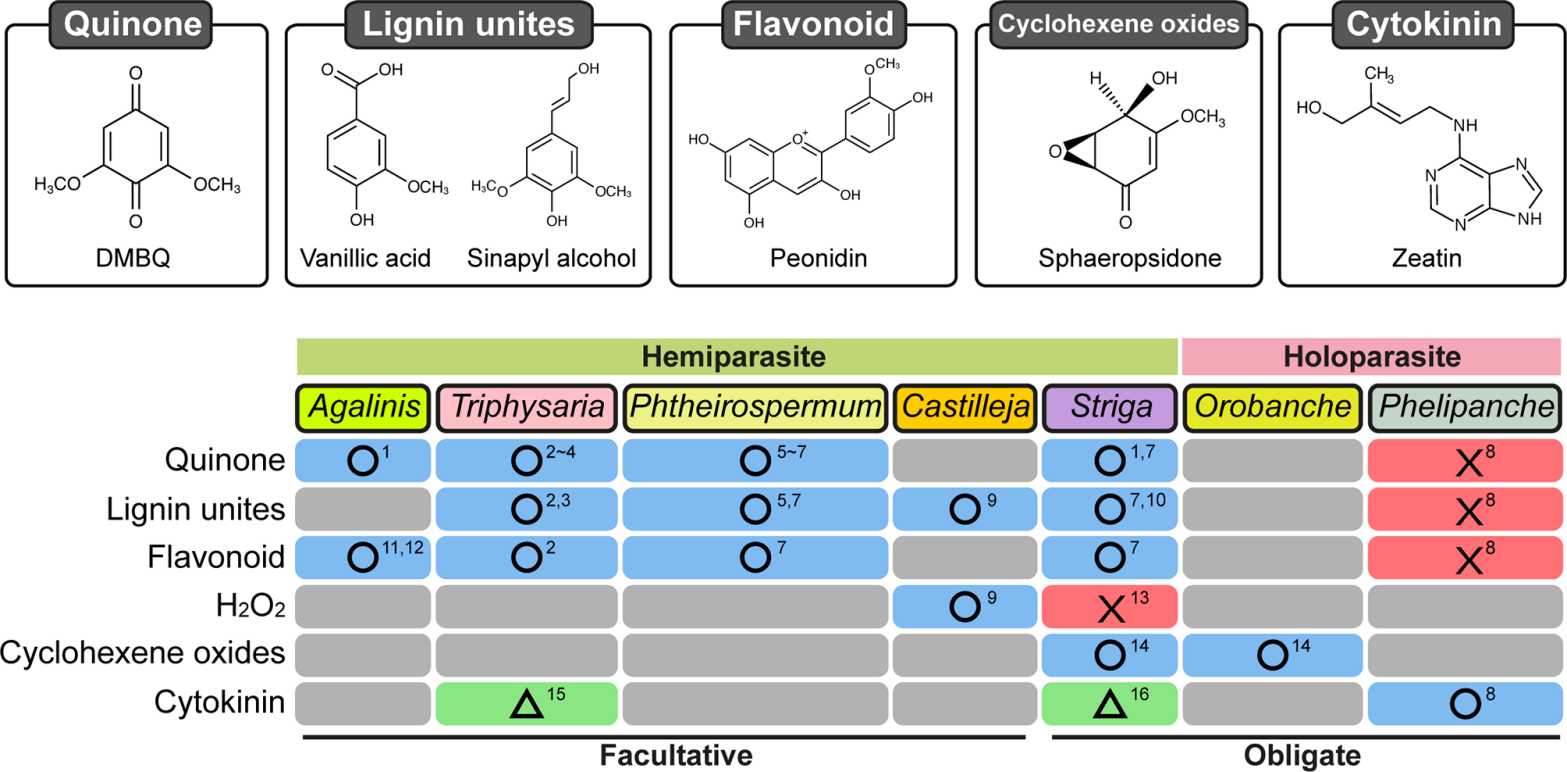
Response of Orobanchaceae parasitic plants treated with various chemicals for haustorium induction. Chemical categories previously reported as HIFs are shown on the left. Blue boxes with circles: at least one chemical was reported in the corresponding species. Red boxes with crosses: tested chemical(s) did not induce haustoria. Green boxes with triangles: haustoria-like structures were reported. Gray boxes: not reported. Numbers indicate references: 1, Chang and Lynn (1986); 2, Albrecht et al. (1999); 3, Bandaranayake et al. (2010); 4, Bandaranayake et al. (2012); 5, Cui et al. (2016); 6, Ishida et al. (2016); 7, Cui et al. (2018); 8, Goyet et al. (2017); 9, Salcedo-Morales et al. (2013); 10, Lynn and Chang (1990); 11, Lynn et al. (1981); 12, Steffens et al. (1982); 13, Wada et al. (2019); 14, Fernández-Aparicio et al. (2016b); 15, Wrobel and Yoder (2001); 16, Keyes et al. (2000).

Induction of prehaustoria by host-derived HIFs is an initial step for haustorium formation. Isolation of HIFs has been accomplished mainly using photosynthetic hemiparasitic species whose prehaustoria are easy to observe due to their characteristic shape and their proliferation of haustorial hairs. HIF activities have been recognized in plant exudates since the 1970s from studies showing that root exudates of numerous plant species were able to induce prehaustoria in the facultative parasites *Agalinis purpurae* and *Castilleja exserta* (previously known as *Orthocarpus purpurascensi*) (Atsatt et al., 1978; Riopel and Musselman, 1979). Several small molecules were afterwards reported to be HIFs (**Figure 2**). Two flavonoids, named xenognosin A and B, were isolated from gum tragacanth, an exudate of *Astragalus* spp., as HIFs for *Agalinis* (Lynn et al., 1981; Steffens et al., 1982). Further attempts to identify HIFs yielded a terpenoid soyasapogenol B from root exudates of *Lespedeza sericea* (Fabaceae), a host of *Agalinis* (Steffens et al., 1983); however, the activity was not sufficiently high to account for the entire host exudate activity (Lynn, 1985; Steffens et al., 1986). A few years later, a quinone 2,6-dimethoxy-1,4-benzoquinone (DMBQ) was isolated from root extracts of sorghum, a natural host for *Striga*, and found to be a HIF for *Striga asiatica* and *Agalinis* (Chang and Lynn, 1986). As DMBQ could not be detected from a healthy root exudate in that report, it has remained questionable whether DMBQ is a naturally occurring HIF. Nevertheless, DMBQ has the highest activity and widest parasite range among currently known HIFs. For example, DMBQ is able to induce haustoria in the obligate hemiparasite *Striga* spp., as well as in the facultative parasites *P. japonicum, Triphysaria* spp., and *Agalinis* spp.(Chang and Lynn, 1986; Ishida et al., 2011; Tomilov et al., 2004). Compounds with structures similar to DMBQ, such as phenolic acids (including syringic acid, vanillic acid, and ferulic acid), aldehydes (including syringaldehyde), and flavonoids (including peonidin), were reported also to induce haustoria in *Triphysaria versicolor*, *P. japonicum*, and *Striga hermonthica* (Albrecht et al., 1999; Cui et al., 2018). *Castilleja tenuiflora* was reported to react with vanillic acid as well as catechin and hydrogen peroxide (H_2_O_2_) (Salcedo-Morales et al., 2013), although H_2_O_2_ alone does not induce haustoria in *S. hermonthica* (Wada et al., 2019).

Because highly active HIFs in host exudates have yet to be identified, localization of HIF production and mode of action of HIFs remain largely unknown. Although some reports suggest that HIF activity is related to the pectin fraction of host cell walls (Keyes et al., 2000), the structures of HIFs are more closely related with those of lignin monomers. Lignin is a complex polymer abundant in the secondary cell walls of vascular plants and is composed of aromatic monolignols, i.e., *p*-coumaryl alcohol, coniferyl alcohol, and sinapyl alcohol, derived from the phenylpropanoid pathway (Vanholme et al., 2010). Polymerization of monolignols results in three major types of generic lignin units, p-hydroxyphenyl (H), guaiacyl (G), and syringil (S) units, which respectively contain zero, one, and two methoxy groups on the aromatic rings. A series of lignin precursors and lignin-derived phenolics were tested for their ability to induce haustoria in *S. hermonthica* and *P. japonicum* (Cui et al., 2018). Interestingly, S-type compounds that have two methoxy groups at the 3 and 5 positions and a hydroxyl group at position 4 of the aromatic ring, e.g., sinapaldehyde, synapyl alcohol, syringic acid, and acetosyringone, can induce haustoria in both *S. hermonthica* and *P. japonicum* (Cui et al., 2018). In contrast, G-type compounds that have one methoxy group at the 3 or 5 position and a hydroxyl group at position 4, including ferulic acid, vanillic acid, vanillin, and apocynin, have a high capacity for inducing haustoria in *S. hermonthica* but not in *P. japonicum*. H-type compounds that do not have a methoxy group generally do not show haustorium-inducing activity for either species (Cui et al., 2018). These results suggest that there are certain species specificities for HIF recognition. Consistently, purified natural lignin polymers as well as artificially synthesized lignin polymers composed of only G-units can induce haustoria only in *S. hermonthica*, and those containing G and S units can induce haustoria in both *P. japonicum* and *S. hermonthica* (Cui et al., 2018). Because the prehaustorium-inducing activity of lignin polymers is greatly enhanced by application of white rot fungi-derived laccase, an enzyme that can produce monolignols and quinones *via* oxidative lignin depolymerization (Pollegioni et al., 2015), monomeric phenolics or quinones are more likely to be the active compounds *in vivo* (Cui et al., 2018). These results could also explain the high HIF activity for *Striga* in the pectin fraction from sorghum cell walls (Keyes et al., 2000). The primary cell wall (pectin-rich) fraction from Poaceae species is known to contain abundant amounts of ester-linked ferulic acid, which acts as a HIF for *Striga* (Harris and Trethewey, 2010). Alteration of the lignin monomer composition in a host using genetic modification affects the haustorium-induction activity of *P. japonicum* and *S. hermonthica* (Cui et al., 2018), indicating that HIFs naturally produced in hosts originate at least partly from lignin biosynthesis or degradation pathways, although details of the origin of HIFs need to be further investigated in the future.

## HIFs for Holoparasites

HIFs have been less studied in holoparasitic Orobanchaceae partly because their prehaustorial structures are less apparent than hemiparasites that proliferate haustorial hairs. Prehaustorium induction in *Orobanche* and *Phelipanche* is nevertheless characterized by radicle growth arrest, radicle tip swelling, and extension of epidermal cells to form secretory papillae bearing a host-attachment function similar to that of haustorial hairs in hemiparasites (Baird and Riopel, 1985; Joel and Losner-Goshen, 1994; Fernández-Aparicio et al., 2016a; Fernández-Aparicio et al., 2016b; Goyet et al., 2017). Because a study reported *in vitro* host-independent prehaustorium induction in the species *Orobanche cumana* and *Phelipanche aegyptiaca* (Joel and Losner-Goshen, 1994), prehaustorium induction in holoparasites has been considered for a long time as a host-independent process. However, recent findings in the species *Phelipanche ramosa* demonstrate that root exudates collected from healthy oilseed rape plants induce prehaustorium formation *in vitro* (Goyet et al., 2017). A similar observation has been made for the species *O. cumana* in response to sunflower root exudates (Montiel & Simier, personal communication). These recent findings therefore imply that prehaustorium induction by host-derived chemical signals or molecules occurring in the rhizosphere is a common process in both hemiparasitic and holoparasitic Orobanchaceae.

However, the currently known HIFs for hemiparasites, including DMBQ, syringic acid, and vanillic acids, are inefficient for inducing prehaustoria in *P. ramosa* (Goyet et al., 2017). In addition, DMBQ is also inactive towards *P. aegyptiaca* (Westwood et al., 2010). Recently, the mycotoxins sphaeropsidone and epi-sphaeropsidone, which are cyclohexene oxides isolated from *Diplodia cupressi* (**Figure 2**), the causal agent for cypress (*Cupressus sempervirens* L.) canker (Evidente et al., 1998), were shown to bear HIF activity *in vitro* towards *Orobanche crenata* and *O. cumana* (Fernández-Aparicio et al., 2016a; Fernández-Aparicio et al., 2016b). Moreover, according to structure-activity relationship analyses, structural modifications of sphaeropsidone and epi-sphaeropsidone affect differently prehaustorium induction in *O. crenata* and in *O. cumana* (Fernández-Aparicio et al., 2016b). As reported in the hemiparasitic Orobanchaceae, these findings suggest that certain species specificities for HIF recognition also occur in the holoparasite *Orobanche*. Nevertheless, these compounds are also active *in vitro* towards *Striga* while being structurally different from the currently known HIFs for hemiparasites. Although activity towards *Striga* could be possibly due to the conversion of sphaeropsidone and its derivatives to active 3-methoxyquinones (Fernández-Aparicio et al., 2016b), this eventually suggests that *Orobanche* may have a more strict specificity for HIF compounds than *Striga*.

## Cytokinins As HIFs

Cytokinins are known to be important phytohormones acting in a vast array of plant development processes including root development as well as nodule formation during plant-bacteria symbiosis (Pacifici et al., 2015). Cytokinins were detected in the rhizosphere of many plants including maize, rice, and tomato (Davey and van Staden, 1976; van Staden and Dimalla, 1976; Soejima et al., 1992; Yang et al., 2002; Kirwa et al., 2018), and interestingly, cytokinins are also effective *in vitro* for inducing prehaustorium-like structures in hemiparasites and prehaustoria in holoparasites (**Figure 2**). Kinetin, a synthetic cytokinin, and 6-benzylaminopurine (BAP) were indeed reported to induce prehaustoria-like structures in *S. asiatica* and in *T. versicolor*, respectively (Keyes et al., 2000; Wrobel and Yoder, 2001). However, some morphological differences between phenolic HIF-induced and cytokinin-induced prehaustoria were underlined; e.g., in *T. versicolor*, cytokinin-induced prehaustoria have smaller swelled structures than those induced by DMBQ. All these findings made questionable the involvement of cytokinins in cooperation with phenolic HIFs as prehaustorium inducers for these hemiparasitic species (Estabrook and Yoder, 1998).

However, recent analyses on prehaustorium formation in the holoparasite *P. ramosa* parasitizing oilseed rape showed clear evidences of the existence of a signal carrying both cytokinin and HIF activities in oilseed rape rhizosphere (Goyet et al., 2017). Bioguided ultra-performance liquid chromatography-electrospray tandem mass spectrometry (UPLC-ESI(+)-/MS/MS) analyses showed that oilseed rape root constitutively exudes a cytokinin with dihydrozeatin characteristics. In parallel, cytokinins at physiological levels (10^−7^–10^−8^ M) were effective for prehaustorium induction in *P. ramosa*. Moreover, as expected from its antagonist activity toward cytokinins, auxin treatment prevented prehaustorium formation in response to cytokinins or root exudates. Gene expression analysis after treatment with oilseed rape root exudates or exogenous t/zeatine confirmed that plant hormone-related genes, including cytokinin-related genes (notably *PrRR5*, *PrCKX2* and *PrCKX4*), were up-regulated during prehaustoria induction (Goyet et al., 2017). These findings highlight cytokinins as *bona fide* rhizosphere signals for holoparasites and that the possible roles for hemiparasite prehaustorium induction need to be re-investigated.

## Action Mechanisms of HIFs in Prehaustorium Induction

Most of the currently known phenolic HIFs for hemiparasites contain an aromatic ring with a hydroxyl group at position 4 and one or two methoxy groups at positions 3 and/or 5 (**Figure 2**). Variation of the functional group at position 1 could affect the haustorium induction activity (Cui et al., 2018), probably due to modification of the redox range of the molecules (Smith et al., 1996), but a structural requirement at position 1 is not apparent. Lignin-related HIFs, syringic acid and syringaldehyde, were reported to be oxidized to produce DMBQ in the presence of peroxidase or laccase *in vitro* (Frick et al., 1996; Ibrahim et al., 2013). Kim et al. (1998) identified peroxidase activity in a *Striga* cell wall fraction that is able to convert syringic acid to DMBQ. Furthermore, quinone oxidoreductase (QR) genes were upregulated in the parasite after DMBQ treatment and/or host infection. Two types of QR (QR1 and QR2) were isolated from *T. versicolor*, *P. japonicum*, and *S. hermonthica*. QR1 reduces one electron from quinone and converts it to a semiquinone, whereas QR2 is thought to reduce two electrons from quinone to hydroquinone (Bandaranayake et al., 2010; Ishida et al., 2017). In *T. versicolor*, *QR1* expression is upregulated by DMBQ and host exudates, whereas *QR2* is induced by DMBQ but not by host exudates (Matvienko et al., 2001). In *P. japonicum* and *S. hermonthica*, *QR2* but not *QR1* expression is upregulated upon both DMBQ treatment and host infection (Ishida et al., 2017). Knockdown of *QR1* expression in *T. versicolor* and *QR2* expression in *P. japonicum* resulted in reduced haustoria formation (Bandaranayake et al., 2010; Ishida et al., 2017). Although it is uncertain why the different types of QRs are involved in haustoria formation in different plant species, the likely scenario is that electron transfer by a QR function is crucial for haustorium initiation.

Oxidation-reduction cycles produce reactive oxygen species (ROS), which may act as signals for haustorium induction (Bandaranayake et al., 2010). To investigate the roles of ROS in haustorium formation, pharmacological analyses were conducted. Application of catalase, a scavenger of H_2_O_2_, reduced haustorium induction of *S. asiatica* by syringic acid but not DMBQ, suggesting that H_2_O_2_ is important for conversion of syringic acid to DMBQ (Keyes et al., 2000). However, a similar experiment using *S. hermonthica* showed that catalase reduces haustorium induction by both DMBQ and syringic acid (Wada et al., 2019), indicating that ROS are also necessary downstream of DMBQ. Application of a series of ROS inhibitors and scavengers revealed that reduced nicotinamide adenine dinucleotide phosphate (NADPH) oxidase inhibitors, especially diphenyleneiodonium (DPI), could efficiently reduce haustorium formation in *S. hermonthica* (Wada et al., 2019). NADPH oxidase is an enzyme that produces O^2−^, which is further converted to H_2_O_2_ by the superoxide dismutase (SOD) enzyme. An inhibitor of SOD also reduced haustorium formation, indicating that H_2_O_2_ production plays a pivotal role for prehaustorium induction in response to DMBQ. Furthermore, peroxidase inhibitors also reduced the haustorium formation rates, whereas exogenous application of peroxidase increased the haustorium formation rates (Wada et al., 2019). These results suggest that ROS modulation *via* peroxidase may regulate HIF-derived signaling, but how ROS and peroxidases interact with HIF perception and signal transduction in the context of haustorium initiation remains to be elucidated.

In the holoparasite *P. ramosa*, transcriptomic analysis focusing on time points surrounding prehaustorium formation in response to HIF-containing host root exudates did not reveal any over-representation of ROS-related Gene Ontology (GO) terms. Therefore, involvement of ROS in prehaustorium induction in holoparasites remains obscure.

## Roles of HIFs in Host-Parasitic Plant Interactions After Prehaustorium Induction

The roles of HIFs in other functions during the host plant-parasitic plant interaction besides prehaustorium induction have not been well studied yet. Cytokinins may have roles during invasion by *P. ramosa* because pre-treatment of germinated seeds of *P. ramosa* with cytokinins increased the number of parasites invading host tissues. Furthermore, pre-treatment with PI-55, an inhibitor of cytokinin signaling (Spíchal et al., 2009), reduced the number of invading parasites (Goyet et al., 2017). Increased successful invasion rates, regarded as *P. ramosa* aggressiveness, upon cytokinin treatment could be due to either modification of prehaustoria formation rates or modification of haustorium functioning. In addition, cytokinins have been reported to manipulate host physiology after successful host penetration (Spallek et al., 2017). Upon infection, *trans*-zeatin-type cytokinins accumulate in both *P. japonicum* and *Arabidopsis thaliana*. Analysis of *Arabidopsis* mutants defective in cytokinin biosynthesis indicated that the accumulated cytokinins in host plants were delivered by the parasites (Spallek et al., 2017). Cytokinin accumulation in the host causes hypertrophy of the infected area of the host and is characterized by an increase in vascular diameter and cell number (Spallek et al., 2017). These morphological and physiological changes of host vascular would lead to increased sink strength at haustorium attachment site, which could contribute to nutrient uptake by the parasitic plant.

Many phenolic HIFs are lignin precursors and, therefore, can be incorporated into lignin polymers in the secondary cell walls of hosts or parasitic plants. Lignin is known to be involved in physical defense against various pathogens including bacteria, fungi, plant-parasitic nematodes, and parasitic plants (Goldwasser et al., 1999; Bhuiyan et al., 2009; Miedes et al., 2014; Khanam et al., 2018; Mutuku et al., 2019). Lignin is deposited at the *Striga* infection site in a *Striga*-resistant rice cultivar, and lignin modification in the rice host affects resistance against *Striga* (Mutuku et al., 2019). Thus, phenolic HIFs may be used by hosts for forming a physical barrier against parasitic plants after parasite attack.

Redox cycling between quinones and hydroquinones occurs in the rhizosphere with a great impact on plant-microbe interactions (Taran et al., 2019). Such redox cycling is known to be used as a lignocellulolytic agent by wood-decaying brown rot fungi. A variation of DMBQ, 2,5-dimethoxy-*p*-benzoquinone (2,5-DMBQ) and its reduced form 2,5-dimethoxy-*p*-hydroquinone (DMHQ) were detected in wafers of aspen wood colonized by the brown rot fungus *Serpula lacrymans* (Korripally et al., 2013). Oxidation of DMHQ to 2,5-DMBQ drives a Fenton reaction: H_2_O_2_ + Fe^2+^ + H^+^ → H_2_O + Fe^3+^ + •OH (Suzuki et al., 2006; Korripally et al., 2013). The resulting hydroxyl radical (·OH) is highly active and non-enzymatically deconstructs the lignocellulose structure (Cragg et al., 2015). During intrusion, parasitic members of the Orobanchaceae family need to pass through lignified endodermal cell layers (Yoshida and Shirasu, 2009), and intrusive cells in some species, such as *Striga* spp., can penetrate into lignified host xylem vessels (Dorr, 1997). It is tempting to hypothesize that parasitic plants may employ the Fenton reaction to depolymerize host lignin during invasion, and quinone-type HIFs may act as driving forces of the reaction. Understanding the physiological roles of HIF molecules beyond prehaustorium induction will help us to know why parasitic plants use these molecules as host signals.

## Conclusions

Evidences collected on hemi- and holoparasites indicate a commonality and specificity of HIFs in parasitic members of the Orobanchaceae family. In addition to cytokinins, ROS-producing quinones or phenolics are commonly recognized as HIFs. For a long time, redox cycling has been suggested to manipulate prehaustorium induction, but the detailed mechanisms of this activity are yet to be discovered. Future studies to understand the common and specific signaling pathways from different types of HIFs should reveal how parasitic plants sense the presence of a host and begin their parasitic lifestyles.

## Contribution to the Field Statement

Parasitic plants in the Orobanchaceae family are among world’s most devastating weed pests, yet the molecular processes underlying their parasitism have not been well understood. Parasitic plants develop haustoria, specialized invasive organs, upon recognition of host-derived chemical signals, the so-called haustorium-inducing factors (HIFs). This review summarizes past work and recent advances in the research of HIFs for parasitic plants in the Orobanchaceae family. Recent research efforts have begun to reveal the origins, species specificity, and action mechanisms of HIFs.

## Author Contributions

VG, SW, and SY conceived the paper and drafted the manuscript. TW and SC drew the figures. SC, TW, KS, GM, PS, and SY edited and finalized the manuscript. 

## Funding

This work is partly supported by a PhD fellowship from the French Ministry of Education and Research to VG, and MEXT KAKENHI grants No. 18H02464 and 18H04838 to SY, 15H05959 to KS, and 19K16169 to SC. This study was also partly supported by the International Atomic Energy Agency Research Contract Number 20645.

## Conflict of Interest Statement

The authors declare that the research was conducted in the absence of any commercial or financial relationships that could be construed as a potential conflict of interest.
